# Anaphylaxis caused by butylscopolamine bromide: a case report

**DOI:** 10.1186/s40981-020-00331-w

**Published:** 2020-04-08

**Authors:** Toshie Shiraishi, Mitsuyo Nakamura, Tatsuo Horiuchi, Tomonori Takazawa

**Affiliations:** 1 Department of Anesthesia, Minimally Invasive Surgery Center, Yotsuya Medical Cube, 7-7 Niban-cho, Chiyoda-ku, Tokyo, 102-0084 Japan; 2 Department of Anesthesiology, Gunma University Graduate School of Medicine, 3-39-22 Showa-machi, Maebashi, Gunma, 371-8511 Japan; 3 Intensive Care Unit, Gunma University Hospital, 3-39-15 Showa-machi, Maebashi, Gunma 371-8511 Japan

**Keywords:** Butylscopolamine, Anaphylaxis, skin test

## Abstract

**Background:**

There have been only few reports on butylscopolamine-induced anaphylaxis despite its global usage as an anticholinergic agent for approximately 70 years. We present a case of anaphylaxis caused by butylscopolamine.

**Case presentation:**

A 63-year-old woman underwent gastrointestinal endoscopic examination. She developed facial cyanosis and hypoxia after intravenous administration of butylscopolamine 10 mg, and her blood pressure was unmeasurable. Her hemodynamic condition recovered after a total of 0.6 mg adrenaline and bolus administration of 100 mg hydrocortisone. One hour after the onset of hypotension, both plasma histamine and serum tryptase were remarkably elevated to 271.7 nmol/L and 174 μg/L, respectively. Skin tests performed 47 days after anaphylaxis showed a positive result only for butylscopolamine among the exposed agents, which was confirmed by basophil activation tests using CD203c and CD63 as markers.

**Conclusion:**

Butylscopolamine has the potential to cause severe anaphylaxis; hence, identification of the causative agent is important to prevent recurrence of anaphylaxis.

## Background

Butylscopolamine bromide has been used worldwide in clinical practice to minimize gastrointestinal movement or treat abdominal pain and is included in the World Health Organization model list of essential medicines [1, 2]. It is considered a relatively safe drug, because there have been only four reports describing butylscopolamine bromide-induced hypersensitivity since its introduction in 1951 [3–6]. Here, we describe the case of a 63-year-old woman who developed anaphylactic shock during gastroscopy, which was diagnosed as butylscopolamine bromide-induced anaphylaxis.

The gold standard diagnostic approach for drug-induced anaphylaxis is a skin test; however, there is room for additional in vitro tests, because non-irritant concentrations for skin testing have not been established for all drugs, particularly for drugs with few clinical reports of anaphylaxis, such as butylscopolamine bromide. As a reliable in vitro test to diagnose the causative agent of anaphylaxis, we employed the basophil activation test (BAT), which is known to have a high sensitivity and specificity [7].

In this case, plasma histamine and serum tryptase levels were measured immediately after the incident, confirming the occurrence of anaphylaxis. We then performed skin tests, which led us to suspect butylscopolamine bromide allergy due to a strong skin reaction. Subsequently, a BAT was performed and activated basophils were detected, confirming the diagnosis.

## Case presentation

Written informed consent for publication of this report was obtained from the patient. A 63-year-old woman (height, 158 cm; body weight, 44 kg) was scheduled for gastrointestinal endoscopic examination because she experienced discomfort in the throat and stomach. She had previously undergone gastrointestinal endoscopic examinations 8 and 15 years ago, the records of which showed that she had uneventfully received butylscopolamine bromide at both examinations. Her pre-examination laboratory data were completely normal.

The events that occurred during endoscopic examination are summarized in chronological order, starting from the administration of butylscopolamine bromide (Table 1). After establishing intravenous access, she was pre-medicated with Pronase® (protease from *Streptomyces griseus*) and Gascon® (dimethicone) syrup orally, followed by application of an 8% lidocaine spray on her throat. Her blood pressure (BP), heart rate (HR), and peripheral blood oxygen saturation (SpO_2_) were 95/51 mmHg, 69 bpm, and 97%, respectively, at this time. The examination was initiated after intravenous administration of 10 mg butylscopolamine bromide to minimize gastrointestinal movement. Additionally, 0.2 mg flunitrazepam and 17.5 mg pethidine hydrochloride were administered intravenously for mild sedation. As her HR increased to 144 bpm and SpO_2_ decreased to 82% 4 min after commencement of the examination, 4 L/min oxygen was supplied via her nose. However, her SpO_2_ decreased further to 72% along with facial cyanosis, and her BP became unmeasurable. As the examination was almost completed, 0.2 mg flumazenil was administered. However, even after administration of flumazenil, the patient did not respond to her name being called or gentle shaking, and her SpO_2_ became undetectable and BP remained unmeasurable. Immediately after announcement of a code blue, an anesthesiologist joined the treatment group and performed endotracheal intubation to secure her airway. After intravenous administration of 0.3 mg adrenaline, her BP recovered to 65/30 mmHg and SpO_2_ improved to 90%. She was then transferred to the post-anesthesia care unit.

**Table 1:** Chronological presentation of the events that occurred during endoscopic examination

Elapsed time (min)	Events	Vital signs and laboratory data
− 8	Pronase® (protease from *Streptomyces griseus*) and Gascon® (dimethicone) syrup were given orally as pre-medication.	
− 1	The patient’s throat was sprayed with 8% lidocaine.	HR 69 bpm, BP 96/51 mmHg, SpO_2_ 97%
0	Butylscopolamine bromide 10 mg was administered intravenously to minimize gastrointestinal movement. Flunitrazepam 0.2 mg and pethidine hydrochloride 17.5 mg were given intravenously for mild sedation.	
1	Endoscopic examination commenced.	
6	Since facial cyanosis was observed, 4 L/min oxygen administration was started.	HR 144 bpm, BP 70/42 mmHg, SpO_2_ 82%
8	Endoscopic examination was stopped.	HR 140 bpm, BP unmeasurable, SpO_2_ 72%
10	Flumazenil 0.2 mg was administered and a code blue was called.	HR, SpO_2,_ and BP undetectable
12	The anesthesiologist arrived at the endoscopy center.	
13	Endotracheal intubation was performed. Adrenaline 0.3 mg was administered intravenously.	
19	BP partially recovered and SpO2 transiently improved.	HR 132 bpm, BP 65/30 mmHg, SpO_2_ 90%
27	The patient was transferred to the post-anesthesia care unit.	
28	Additional adrenaline 0.3 mg was administered.	
37	A saline solution was rapidly infused, and hydrocortisone 100 mg was intravenously administered. Spontaneous respiration was assisted with 5 cm H_2_O CPAP at an FiO_2_ of 0.5.	HR 125 bpm, BP 76/47 mmHg, SpO_2_ 94%, PaO_2_ 197 mmHg PaCO_2_ 49 mmHg, BE 0.9 mmol/L
42	Light systemic skin rashes were observed on the patient’s body.	
57	Blood samples were collected to measure plasma histamine and serum tryptase levels.	Histamine 271.7 nmol/L, tryptase 174 μg/L
77	The patient’s trachea was extubated.	HR 110 mmHg, BP 103/44 mmHg, SpO_2_ 99%

*HR* heart rate, *BP* blood pressure, *SpO*_*2*_ peripheral blood oxygen saturation

Her BP and SpO_2_ gradually recovered after additional intravenous administration of 0.3 mg adrenaline, rapid infusion of 500 mL saline, and intravenous administration of 100 mg hydrocortisone. A continuous positive airway pressure of 5 cm H_2_O at an FiO_2_ of 50% was applied to assist her spontaneous respiratory efforts. Arterial gas measurements showed that PaO_2_, PaCO_2_, and base excess were 197 mmHg, 49 mmHg, and 0.9 mmol/L, respectively. Simultaneously, light skin rashes were observed on her body. Therefore, we suspected grade III anaphylaxis and collected a blood sample for measurement of plasma histamine and serum tryptase levels. Her cardiovascular condition stabilized at 2 h after the event and her trachea was extubated. Plasma histamine and serum tryptase levels at 1 h after the onset of shock were markedly elevated to 271.7 nmol/L and 174 μg/L, respectively. She was hospitalized to confirm the absence of relapse of symptoms, including biphasic anaphylaxis, and was discharged the subsequent day as her vital signs had stabilized.

**Fig. 1:**
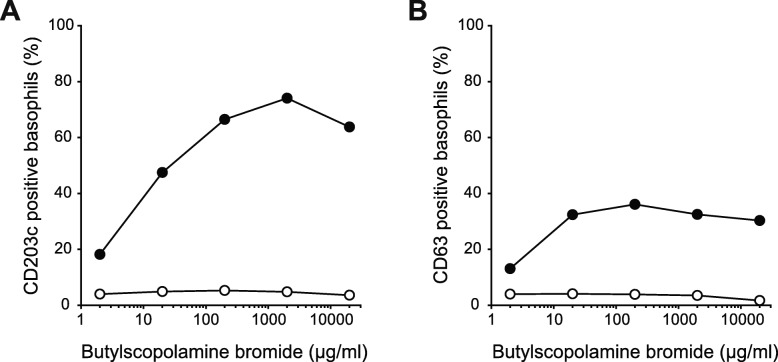
CD203c (**a**) and CD63 (**b**) upregulation in a patient with butylscopolamine bromide-induced anaphylaxis (closed circles) and a control individual (open circles)

Forty-seven days after the event, further examination was scheduled to identify the cause of anaphylaxis. She was informed about the diagnostic examinations and her consent was obtained. All the necessary drugs and equipment required for resuscitation were readily available. All the pharmaceutical agents to which she had been exposed during endoscopic examination were included in the skin test. Skin reactions were considered positive when the wheal diameter increased by 3 mm or more after 20 min [8, 9]. Evaluation showed that skin prick tests were positive only to a 1:100 dilution (100 μg/mL) of butylscopolamine bromide, which produced a 4-mm-diameter wheal, and erythema of 7-mm diameter (Table 2). Subsequently, we collected blood samples to perform the BAT after stimulation with butylscopolamine bromide, as detailed elsewhere [10–12]. Briefly, CD203c and CD63 were used as markers to detect activated basophils using a flow cytometer (FACSCanto II; BD Bioscience, San Jose, CA). The patient’s ratio of activated basophils was calculated and compared with that of a healthy male volunteer with no allergic skin test reaction to butylscopolamine bromide. The patient, but not the volunteer, demonstrated dose-dependent upregulation of CD203c and CD63 (Fig. 1). The patient’s plasma histamine and serum tryptase levels were evaluated again on the same day as the BAT (47 days after the event) and were found to have returned to normal levels (6.3 nmol/mL and 2.1 μg/L, respectively).

**Table 2:** Results of skin prick tests

Drug	Concentration of the stock solution (mg/mL)	Judgment	Wheal (mm)	Flare (mm)
Histamine	NA	+	3	3
Saline	NA	-		
Lidocaine	10	-		
Flunitrazepam	2	-		
Butylscopolamine	10	+	4	7
Dimethicone	20	-		
Pethidine	50	-		
Pronase	10	-		

Histamine (10 mg/mL) and saline were used as positive and negative controls, respectively. For other drugs, we used a test solution in which the stock solution was diluted 100 times

## Discussion

Since the first case report in 1982, there are only four published reports of butylscopolamine bromide-induced allergic reactions [3–6]. Although one of them was fatal, the remaining three were mild reactions, with the appearance of only skin symptoms. In one case, the diagnosis was confirmed with skin prick and oral challenge tests, although such tests were not performed in the other cases, including the one that was fatal. The Japanese database on adverse drug reactions managed by the Pharmaceuticals and Medical Devices Agency contains information on 70 suspected cases of butylscopolamine bromide-induced hypersensitivity, but the available information is limited [13]. Among these, 20 cases of anaphylaxis during gastrointestinal examinations, as in our case, have been reported. Although there were three fatal cases due to anaphylaxis, detailed information on these cases is not available [13]. Thus, there are only a few cases with a definitive diagnosis, even in those with severe anaphylaxis.

Of the several inflammatory mediators that are released in anaphylaxis, tryptase is most widely assessed in blood tests [14]. In our case, we found a marked increase in serum tryptase at 1 h after the reaction (174 μg/L) compared to the baseline level (2.1 μg/L). Since an increase in serum tryptase at the time of a suspected reaction to above 1.2 × baseline + 2 μg/L is considered clinically relevant [15], the results suggested the occurrence of a strong hypersensitivity reaction. Significant elevation of plasma histamine levels at 1 h after the reaction (271.7 nmol/L) compared to baseline levels (6.3 nmol/mL) also supported our diagnosis.

Although allergic reactions to butylscopolamine bromide are not rare [13], the diagnostic method has not been corroborated. In this case, we obtained a positive reaction in skin prick tests with 100 μg/mL butylscopolamine bromide, which is 200 times lower than a previously reported test concentration (20 mg/mL) [6]. It is known that the higher the drug concentration, the higher the false-positive rate; therefore, the test result in this case was considered most likely a true positive. Furthermore, the BAT was conducted in addition to the skin prick test; the results showed much higher basophil activation rates in the patient than those in a control (Fig. 1). Although it would be better to determine the maximum drug concentration that does not show false positive results in skin tests and the threshold to distinguish positive/negative responses for BATs, this is not always possible for drugs with only few clinical reports. As skin tests are recommended to be performed 4–6 weeks after the anaphylactic incident, the interval was too short to determine the optimal drug concentration for these skin tests [7]. Therefore, in the future, it is necessary to verify the optimal drug concentration needed for skin testing with butylscopolamine bromide.

We made a definitive diagnosis of butylscopolamine bromide-induced anaphylaxis using a combination of skin tests and BATs. Skin prick tests are the standard procedure to identify causative agents of allergy, but simultaneously performing the BAT strengthens the diagnosis by corroborating the results of skin tests, especially for drugs whose recommended concentrations for skin tests are unknown and are not listed in the guidelines.

Further, drug allergy reports should be made more open to the public to increase awareness about potential hypersensitivity. Our experience highlights the fact that any drug can cause severe anaphylaxis and that identification of the causative agent of anaphylaxis is important to prevent recurrence.

## Data Availability

Data relevant to this case report are not available for public access because of patient privacy concerns but are available from the corresponding author on reasonable request.

## Abbreviations

BAT: Basophil activation test
BP: Blood pressure
HR: Heart rate
SpO_2_: Peripheral oxygen saturation
